# A randomized controlled trial of potential tobacco policies prohibiting menthol flavor in cigarettes and e-cigarettes: a study protocol

**DOI:** 10.1186/s12888-024-05619-0

**Published:** 2024-03-12

**Authors:** Krysten W. Bold, Akshika Sharma, Angela Haeny, Ralitza Gueorguieva, Eugenia Buta, Stephen Baldassarri, Lauren Lempert, Suchitra Krishnan-Sarin, Stephanie O’Malley

**Affiliations:** 1 Yale School of Medicine, Department of Psychiatry, New Haven, CT USA; 2 Yale Cancer Center, New Haven, CT USA; 3 Department of Biostatistics, Yale School of Public Health, New Haven, CT USA; 4 Yale Center for Analytical Sciences, Department of Biostatistics, Yale School of Public Health, New Haven, CT USA; 5 Department of Internal Medicine, Section of Pulmonary, Critical Care, and Sleep Medicine, Yale School of Medicine, New Haven, CT USA; 6 Program in Addiction Medicine, Yale School of Medicine, New Haven, CT USA; 7 Department of Pediatrics, Division of Adolescent Medicine, Stanford University, Palo Alto, CT USA; 8 Center for Tobacco Control Research and Education, University of California, San Francisco, CA USA

**Keywords:** tobacco regulatory science, e-cigarette, cigarette, menthol ban, policy

## Abstract

**Background:**

Menthol cigarette use remains a large public health problem and disproportionately affects Black adults in the United States. The Food and Drug Administration has proposed prohibiting menthol flavor in cigarettes to protect public health. However, e-cigarettes are available in menthol flavor and are a popular alternative product adults might switch to if menthol is prohibited in cigarettes. Research is needed to understand how availability of menthol (vs. tobacco) flavored e-cigarettes could impact cigarette use among adults who smoke menthol cigarettes.

**Methods:**

We will recruit 150 adults who currently smoke menthol cigarettes and will randomize them to 1 of 3 conditions modeling different regulatory scenarios. We will recruit equal numbers of participants identifying as Black vs. non-Black and will stratify randomization by race. To promote standardization and adherence, cigarette and e-cigarette products will be provided for 8 weeks based on the assigned condition: (A) no menthol restriction (menthol cigarette and menthol flavored e-cigarette), (B) menthol prohibited in cigarettes only (non-menthol cigarette and menthol flavored e-cigarette), (C) menthol prohibited in both cigarettes and e-cigarettes (non-menthol cigarette and tobacco flavored e-cigarette). A follow-up visit will occur at week 12 to assess tobacco use status. The study aims are to (1) examine the impact of prohibiting menthol flavor in cigarettes and e-cigarettes on smoking behavior and (2) investigate whether outcomes differ by race to understand the impact of menthol policies on Black (vs. non-Black) individuals given high rates of menthol cigarette use in this population. The primary outcome will evaluate changes in the number of cigarettes smoked per day during the 8-week study period and will examine differences by regulatory scenario. Secondary outcomes will compare percent days smoke-free, changes in nicotine dependence, and motivation, confidence, and intentions to quit smoking by the regulatory scenarios. We will examine whether changes in the outcomes differ by Black vs. non-Black participants to compare the magnitude of the effect of the various menthol policy scenarios by race.

**Discussion:**

Results will contribute critical information regarding menthol in cigarettes and e-cigarettes to inform regulatory policies that maximize reductions in cigarette smoking and reduce tobacco-related health disparities.

**Trial registration:**

NCT05259566. Yale IRB protocol #2000032211, last approved 12/8/2023.

## Background

Cigarette smoking remains the leading cause of preventable death worldwide [1, 2]. Although the overall rate of cigarette smoking among adults has declined in recent years, menthol cigarette use has not decreased at the same rate as non-menthol cigarette use [3, 4], and menthol cigarette use remains a serious public health problem. Use of menthol cigarettes is associated with greater nicotine dependence and lower rates of successful cessation [5, 6]. Furthermore, rates of menthol cigarette use in the United States are highest among Black people [4], contributing to tobacco-related health disparities in this population [7]. More focused and rigorous tobacco control policies are needed to address menthol cigarette use.

The World Health Organization has recommended prohibiting menthol and other flavors in cigarettes [8], and these policies have been enacted in several countries worldwide [9, 10]. In the United States, the Food and Drug Administration (FDA) has the authority to regulate the manufacturing, marketing, and distribution of tobacco products. When the US Tobacco Control Act prohibited the sale of cigarettes with characterizing flavors in 2009, menthol cigarettes were exempt. In May 2022, the FDA proposed a product standard to prohibit menthol as a characterizing flavor in cigarettes [11], although the timeline of enforcement is currently unknown.

Strong scientific evidence is essential for supporting these regulatory policies, and accumulating evidence indicates prohibiting menthol flavor in cigarettes would benefit public health [12, 13]. Studies have assessed the potential impact of this policy on smoking behavior using surveys [14, 15], population-level simulations [16], and observations from other countries that have already enacted a policy prohibiting menthol in cigarettes [15, 17]. Findings indicate this policy would reduce cigarette use and increase quit attempts [18]. To provide further real-world empirical evidence on the effects of prohibiting menthol cigarettes, we developed and tested a within-person product-switching clinical research paradigm, wherein adults who smoke menthol cigarettes are switched to non-menthol cigarettes to test the effects on real-world smoking behavior. Our pilot work indicated that after switching from menthol to non-menthol cigarettes, individuals smoked fewer cigarettes per day, had lower nicotine dependence, and greater motivation and confidence to quit smoking [19]. Moreover, initial findings by race indicated greater reductions in smoking among Black vs. non-Black participants [19]. These results provide further support for tobacco regulatory policies prohibiting menthol cigarettes and indicate such policies may reduce cigarette use and improve public health.

To date, the FDA has proposed product standards on combustible tobacco products (i.e., prohibiting characterizing menthol flavor in cigarettes and all flavors in cigars [11, 20]) to reduce appeal and use, but has not extended this policy to restrict menthol flavors in non-combustible products including e-cigarettes. Additional research is needed to inform evidence-based policies for menthol flavor in e-cigarettes. Following the Deeming Rule in 2016, the FDA now has the authority to regulate the flavors available in e-cigarettes [21]. The FDA is currently reviewing the evidence on flavored e-cigarettes and has published guidance prioritizing enforcement against the sale of e-cigarette flavors that are popular among youth (e.g., fruit, candy) in cartridge-based (“pod”) devices, but allowing for the continued sale of tobacco and menthol flavored e-cigarettes [22, 23]. However, few studies have examined the role of e-cigarette flavors in pod devices on smoking behavior among adults [24].

E-cigarettes are the most commonly used non-cigarette tobacco product among adults, and over 11 million US adults currently use e-cigarettes [25]. Thus, e-cigarettes are a common alternative tobacco product that adults might switch to as a replacement for cigarettes in the context of a policy prohibiting menthol cigarettes. Additionally, pod devices have more efficient nicotine delivery than earlier generation e-cigarettes, and may be a better substitute for cigarettes for adults who smoke [26, 27]. There is evidence that switching completely from combustible cigarettes to e-cigarettes has the potential to encourage cessation of combustible cigarettes and reduce exposure to toxicants [28, 29]. Furthermore, studies suggest that adults who smoke menthol cigarettes (vs. those who smoke non-menthol) are more likely to use e-cigarettes [30], potentially as a way to stop or reduce cigarette smoking [31], and a pilot study modeling a menthol cigarette ban indicates that adults who smoke menthol cigarettes may select e-cigarettes in conditions where menthol is banned in cigarettes [32]. Thus, research is needed to investigate the role of e-cigarette flavors in the context of a menthol cigarette ban to better understand the impact of tobacco and menthol flavored e-cigarettes on smoking behavior.

The current study will provide new information by modeling the effect of potential policies prohibiting characterizing menthol flavor in cigarettes, e-cigarettes, or both products and will examine the impact on real-world smoking behavior. We will adapt our product-switching paradigm and randomize adults who smoke menthol cigarettes to 1 of 3 groups for 8 weeks of product use to model the impact of three different regulatory scenarios: (A) no menthol restriction (usual menthol cigarettes and menthol flavored e-cigarette available), (B) menthol prohibited in cigarettes only (non-menthol cigarettes and menthol flavored e-cigarette available), and (C) menthol prohibited in both cigarettes and e-cigarettes (non-menthol cigarettes and tobacco flavored e-cigarette available). An 8-week exposure to the possible regulatory scenarios was selected to be consistent with other seminal tobacco regulatory studies that evaluated effects of switching to an alternative tobacco product for 6–8 weeks [33–35]. Significant changes in smoking behavior in these studies were evident 2–4 weeks after switching to an alternative tobacco product [33–35], consistent with the results observed in our initial study [19]. Thus, 8-weeks of exposure will allow us to examine the durability of these initial changes.

Aim 1 of the study will examine the impact of prohibiting menthol flavor in cigarettes and e-cigarettes on smoking behavior. The primary outcome will evaluate changes in the number of cigarettes smoked per day during the 8-week exposure to the regulatory scenarios. We hypothesize (1) greater reductions in cigarette use in the scenarios prohibiting menthol cigarettes (groups B/C) vs. no menthol restriction (group A) and (2) the greatest reductions in cigarettes per day where menthol is prohibited in cigarettes but where menthol e-cigarettes are available as a substitute (group B) compared with groups A and C. Secondary outcomes will compare percent days smoke-free, changes in nicotine dependence, and motivation, confidence, and intentions to quit smoking across the regulatory scenarios. Exploratory outcomes will examine percent days of e-cigarette use during the 8-week period as well as continued product use and quit attempts at a follow-up visit at week 12. Aim 2 will investigate whether outcomes differ by race to understand the impact of the menthol policies on Black vs. non-Black individuals given high rates of menthol cigarette use among Black adults. This project will provide critical information to understand whether continued availability of menthol (vs. tobacco) flavor in e-cigarettes will lead to greater reductions in cigarette use and complete switching in the context of a policy prohibiting menthol cigarettes. Findings will provide timely and critical evidence to inform regulatory actions regarding menthol flavor in cigarettes and e-cigarettes.

## Methods

### Study design

A randomized controlled trial funded by the National Institute on Drug Abuse of the National Institutes of Health will be conducted (R01DA054993). Participants will be allocated prospectively to 1 of 3 conditions.

### Ethics approval

Ethical approval for the human subjects procedures of this trial was obtained from the Yale University Institutional Review Board Human Investigation Committee (HIC approval #2000032211. The Yale IRB will conduct continuing review of the protocol every 6 months. Any protocol modifications will be submitted to the Yale IRB for review prior to implementation.

### Participants

We will recruit 150 adults (21 years or older) from the community around New Haven, Connecticut, who currently smoke menthol cigarettes. We will use recruitment strategies including flyers, online and print advertisements, and word-of-mouth. Additionally, we will partner with the Yale Center for Clinical Investigation (YCCI) cultural ambassadors that serve as liaisons to the community to support community-based recruitment for research projects. We aim to recruit equal numbers of participants who identify as Black vs. non-Black and will stratify randomization by race to ensure equivalent representation across conditions. Every effort will be made to recruit equal numbers of sexes, and we will compare responses between sexes as appropriate for each aim.

**Eligibility criteria**: Inclusion Criteria: (1) age 21 or older, (2) report smoking at least 5 cigarettes per day for at least the past 6 months, (3) report currently smoking menthol cigarettes, (4) baseline tobacco use status biochemically confirmed with urine cotinine levels (> 200ng/ml), (5) willing to try e-cigarettes, (6) English speaking, (7) willing to complete study procedures.

Exclusion Criteria: (1) seeking smoking cessation treatment or currently trying to quit smoking; (2) reporting a serious, unstable psychiatric condition (e.g., bipolar disorder, schizophrenia), (3) reporting a serious, unstable physical health condition that would increase risk of study participation determined by medical history interview and review by study physician (e.g., past or present cardiac problems, respiratory problems, overnight hospitalization in the past 3 months), (4) current criteria for DSM-5 moderate or severe cannabis use disorder, (5) current use of other psychoactive substances confirmed by urine drug screening, unless prescribed and stable for 2 months, (6) living with someone who currently smokes menthol cigarettes, to reduce access to preferred cigarettes during the switching phase of the study, consistent with our pilot [19], (7) known allergy to PG/VG in e-liquid, (8) female participants will be excluded if they are currently pregnant, planning to become pregnant, breastfeeding, or unwilling to use an effective method of birth control for the duration of the study, (9) abnormal spirometry test, (10) if reporting recent cannabis vaping (past 90 days), additional exclusion will be any report of mild or greater EVALI-related symptoms (e.g., cough, shortness of breath, chest pain) without non-EVALI reasonable and proximal cause.

### Procedures

#### Screening and consent

Interested participants who contact the research team by telephone or email will have the option to complete a brief pre-screening survey by phone or online. Before completing the pre-screening survey, participants will provide informed consent to complete the screening process. At each stage of the screening, participants will have the opportunity to ask questions. Participants will be told this is not a treatment study, and if they wish to quit tobacco use at any point during the screening process or study participation, cessation referral resources will be provided to them. Following completion of the pre-screener, potential participants who meet initial eligibility criteria will be invited to complete an intake appointment with the research assistant who will obtain written informed consent for study participation and screen for inclusion/exclusion criteria. Table 1 presents the study assessments by timepoint. Following informed consent, intake assessments will be administered to screen for eligibility. Assessments include (1) demographic information, (2) smoking history and a timeline-follow-back interview to assess tobacco use including smoking quantity and frequency such as number of cigarettes per day and number of years smoked, (3) expired breath carbon monoxide (CO) measured in parts per million, (4) a urine sample for screening for cotinine, pregnancy, and recent drug use, and (5) a health interview to rule out high-risk participants with unstable conditions. Eligible individuals who consent to participate will complete the following study activities described below.

**Table 1:** Study assessments

TIMEPOINT	Intake	Week − 1	Week 0	Week 2	Week 4	Week 6	Week 8	Week 12
**Enrollment**								
Eligibility screening	x							
Informed Consent	x							
Randomization			x					
**Interventions**								
Usual brand menthol cigarettes available		x						
Cigarettes and e-cigarettes available by regulatory scenario: (A) no menthol ban, (B) menthol ban in cigarettes only, or (C) menthol ban in both cigarettes and e-cigarettes			x	x	x	x	x	
**Assessments**								
Demographics	x							
Tobacco use history	x							
Medical history interview	x							
Mood (anxiety GAD-7^a^, depression PHQ-8^b^)		x						
Quality of life^c^		x					x	
Racial trauma and discrimination (TSDS^d^) and ethnic identity (MEIM^e^)		x						
Timeline follow-back interview^f^ (cigarettes, e-cigarettes, cannabis and other tobacco products)	x	x	x	x	x	x	x	x
Nicotine dependence (cigarette and e-cigarette PROMIS measures)^g,h^			x		x		x	x
Wisconsin Inventory of Smoking Dependence Motives (WISDM-37)i		x					x	
Time to first cigarette (FTND)^j^		x	x		x		x	x
Cigarette quitting motivation and confidence^k^			x				x	x
Product satisfaction (Modified cigarette evaluation scale, modified e-cigarette evaluation scale)^l,m^			x	x	x	x	x	
Product craving^n^ (cigarette, e-cigarette)			x	x	x	x	x	x
Nicotine withdrawal (Minnesota Nicotine Withdrawal Scale^o^)			x	x	x	x	x	x
Health and safety assessment		x	x	x	x	x	x	x
Protocol adherence measures (spent cigarette filters, e-cigarette pods, use of other mentholated products)		x	x	x	x	x	x	x
**Biochemical measures**								
Urine pregnancy test	x	x	x	x	x	x	x	
Urine biomarkers (cotinine, total nicotine equivalents, menthol glucuronide)	x		x		x		x	
Expired breath carbon monoxide (CO)	x	x	x	x	x	x	x	x
Spirometer lung function test	x	x	x	x	x	x	x	

Footnotes:

^a^Generalized Anxiety Disorder-7 (GAD-7) [47]; ^b^Patient Health Questionnaire-8 (PHQ-8): *8-item version adapted from the PHQ-9* [48]; ^c^Quality of Life [49]; ^d^Trauma Symptoms of Discrimination Scale (TSDS) [50]; ^e^Multigroup Ethnic Identity Measure (MEIM) [51, 52]; ^f^Timeline Follow Back Interview (TLFB) [42]; ^g^PROMIS measure of nicotine dependence [53]; ^h^PROMIS measure of e-cigarette dependence [45]; ^i^Wisconsin Inventory of Smoking Dependence Motives (WISDM-37) [54, 55]; ^j^Fagerstrom Test for Nicotine Dependence (FTND) [56]; ^k^Quitting motivation and confidence: *Rated from 1 (not at all) to 10 (extremely) from the PhenX toolkit*; ^l^Modified Cigarette Evaluation Questionnaire (MCEQ) [57]; ^m^Modified E-Cigarette Evaluation Questionnaire (MECEQ) [58]; ^n^Craving measured with 2-items from the Mood and Physical Symptoms Scale (MPSS) [59]; ^o^Minnesota Nicotine Withdrawal Scale (MNWS) [60]

#### Randomization

Participants will be randomized to 1 of 3 study conditions modeling different regulatory scenarios using stratified block randomization. We will use a randomization ratio of 1:1:1 to assign *N* = 50 per condition. The study biostatistician will create the randomization list that will be stratified by smoking heaviness (≤ 10 or > 10 cigarettes per day) and race (Black vs. non-Black) to ensure equivalent representation across groups. To avoid randomized non-starters and potential bias due to differential early attrition from prior knowledge of group assignment, randomization will occur at the time of visit 0 when participants first receive tobacco products specific to their assigned condition, and neither the research assistant nor participant will know the assignment in advance.

#### Visit schedule

**Fig. 1:**
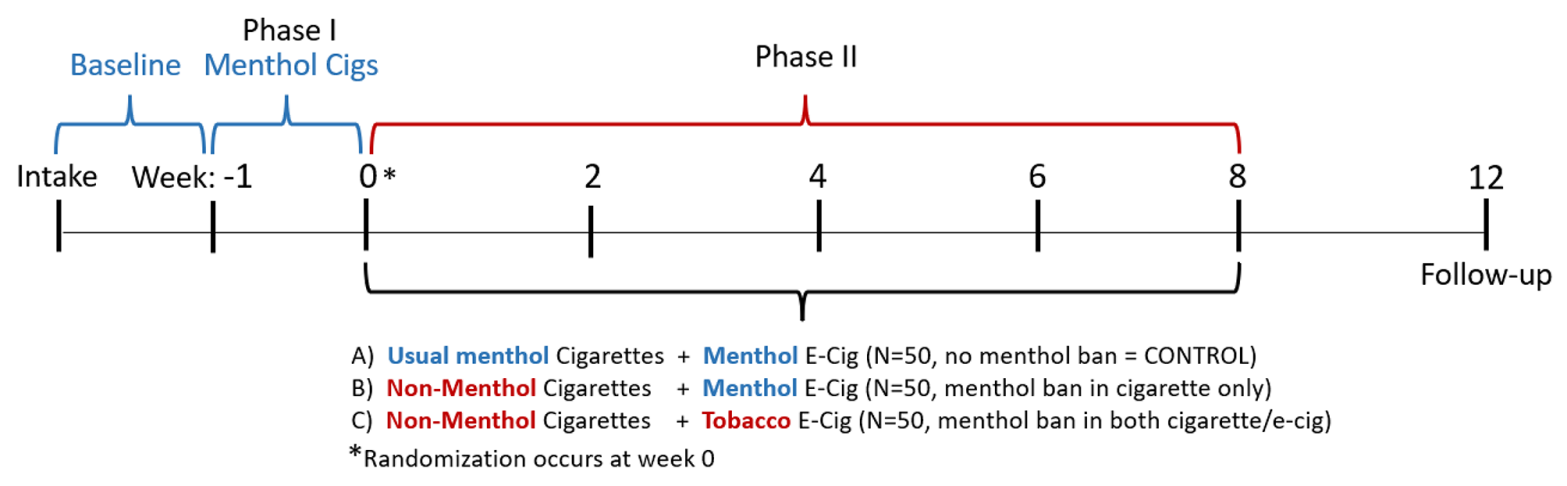
Study design

Following the intake, enrolled participants will complete research assessments at week − 1, 0, 2, 4, 6, 8, and 12. To promote standardization and adherence, cigarette and e-cigarette products will be provided for the study duration based on the assigned condition. At week − 1, all participants will be provided with their usual brand menthol cigarettes to smoke for one week (Phase I) and then will receive tobacco products in line with their assigned regulatory scenario for 8 weeks (Phase II) (see Fig. 1 for study timeline). Providing cigarette and e-cigarette products in both phases encourages adherence to the assigned study condition and controls for the possibility that free products might influence use (as seen in other studies [34]). This way, cost of cigarettes and e-cigarettes is removed as a factor that can potentially influence differences in smoking behavior from Phase I to Phase II.

#### Product use instructions and safety monitoring

Participants will be told to only use the tobacco products we provide during the study. All participants will receive standardized instructions on how to use the e-cigarette. Participants will be educated on the device features and will complete 3 observed puffs when they first receive the e-cigarette to ensure everyone has tried the device and knows how it works. Further, individuals will be encouraged to try the e-cigarette during the 8-week ad-lib period as a substitute for cigarettes. We will inform participants about the potential risks of dual use of cigarettes and e-cigarettes and will discuss possible signs of too much nicotine exposure (e.g., nausea, vomiting, dizziness). We will instruct participants to reduce their cigarette and/or e-cigarette use if they experience any of these symptoms. A standardized assessment of health symptoms will be assessed at each visit and any adverse events will be documented. In consultation with the study physician, research staff may switch participants to a lower nicotine concentration e-cigarette (3% instead of 5%) if necessary, in response to adverse events. If an adverse event occurs, the PI will review and determine the attribution and grade of severity. While serious adverse events (SAEs) or other problems are not anticipated, serious and unanticipated and related adverse events or unanticipated problems involving risks to participants or others will be reported within 48 h to the Yale IRB and within 72 h to the relevant Serious Adverse Event Tracking and Reporting System at NIDA/NIH.

#### Product adherence

We will collect any remaining cigarettes or e-cigarettes from participants prior to giving study products. We will provide participants with instructions on how to save and return spent cigarette filters and e-cigarette pods so the count of used product can be verified by research staff, procedures that are well-established and have been utilized previously to verify adherence [19, 36]. Participants will be incentivized to return the spent cigarette filters and used/unused e-cigarette pods with a possible bonus payment each week. Importantly, participants are paid this bonus for returning the spent cigarette filters/e-cigarette pods regardless of adherence to the assigned condition to encourage honest reporting, which was useful for obtaining accurate measures of adherence in our pilot [19].

#### End of study

At the week 8 study visit, we will have participants return any unused cigarettes, the e-cigarette device, and any unused e-cigarette pods. We do this both so that we can properly dispose of the products and so we can quantify use by counting used/unused cigarettes/pods. We will incentivize returning the products with a bonus payment, so in the case that a participant has stopped cigarette use completely and desires to continue using e-cigarette to prevent a return to cigarette smoking, the study earnings can be used to purchase their own device. Although this is not a treatment study, we will provide all participants with a list of tobacco cessation resources at the end of the study and provide a brief motivational interviewing session to review the benefits of stopping smoking. If participants are using both products, we will discuss potential harms of dual use of cigarettes and e-cigarettes and advise them to stop cigarette use completely and seek additional evidence-based support to stop smoking. We will conduct a brief follow-up survey 4 weeks later (week 12) to assess tobacco use outcomes following exposure to the regulatory scenario once tobacco products are no longer provided.

#### Retention

We will promote participant retention by providing compensation at each visit and providing options to meet at a convenient location for the participant or providing free parking and payment for travel to the office. We will maintain consistent contact via email, text message and phone calls from enrollment through follow-up study visits, and participants will receive reminders in advance of their study visits. We will emphasize the importance of follow-up and ask participants to inform of us of any changes to their contact information over the course of the study. At the start of the study, we will also request contact information for two people who always know how to get in touch with the participant to use only in the event that the participant cannot be contacted at follow-up time points. These sources are used to obtain forwarding addresses or phone numbers in the event that contact information for a participant has changed. Furthermore, if necessary, we have established procedures for obtaining outcome data on cigarette and e-cigarette use and survey information remotely via phone, secure Zoom video conferencing, and secure Yale Qualtrics survey links.

### Interventions

A. **No menthol restriction**: Participants randomized to the regulatory scenario with no menthol restriction will continue to receive their usual menthol cigarettes and a menthol-flavored e-cigarette. This was selected as a control condition because it models the current status quo scenario where there is no prohibition of menthol in either product. This design also allows for consistency across conditions since all participants will have access to both cigarettes and e-cigarette products with the primary difference being the flavor of the products. We selected commercially-available pod e-cigarettes in 5% nicotine salt concentration for this study for several reasons. These are the most popular e-cigarettes on the market [37, 38], and they have a nicotine pharmacokinetic profile like that of combustible cigarettes with more favorable subjective ratings than other e-cigarettes [27, 39], which may make them a more acceptable substitute for adults who smoke cigarettes. Additionally, pod e-cigarette devices have design features that enhance ease of use, consistency of experience, and safety for the user. These e-cigarettes are small, do not require knowledge of modifying temperature/voltage controls, and are a closed system, meaning the device and e-liquids are not modifiable by the participant and it does not require refilling e-liquid cartridges.
B. **Menthol prohibited in cigarettes**: Participants randomized to the regulatory scenario with menthol flavor prohibited in cigarettes but not e-cigarettes will be switched from their usual menthol cigarettes to replacement non-menthol cigarettes of the same brand (e.g., switching from Newport menthol to Newport non-menthol) and will receive a menthol-flavored e-cigarette. A matched-brand non-menthol cigarette comparison was selected based on real-world evidence from a menthol ban in Canada where it was observed that the tobacco industry encouraged consumers to maintain brand loyalty and switch to their same-brand non-menthol products after the ban [40, 41].
C. **Menthol prohibited in cigarettes and e-cigarettes**: Participants randomized to the regulatory scenario with menthol flavor prohibited in cigarettes and e-cigarettes will receive matched-brand non-menthol cigarettes and a tobacco flavored e-cigarette.

### Measurements

#### Primary outcome measure

The primary outcome measure is changes in cigarettes smoked per day. At each assessment, participants will report the total number of cigarettes consumed each day and the total number of times the e-cigarette is used each day using well-validated timeline follow-back (TLFB) interview methods [42]. The primary outcome will examine the average number of cigarettes smoked per day in the past week at visit 0, 2, 4, 6, and 8 to evaluate changes in smoking behavior by regulatory scenario. Additional outcomes will assess the percent days smoke-free and percent days of e-cigarette use during the 8-week period.

#### Secondary outcomes

Secondary outcomes will compare percent days smoke-free measured via TLFB assessments. Additional secondary outcomes include changes in nicotine dependence measured by the PROMIS® measure of cigarette dependence and a corresponding measure of e-cigarette dependence [43–45], and motivation, confidence, and intentions to quit smoking [46] among the regulatory scenarios.

#### Exploratory outcomes

Exploratory outcomes will examine percent days of e-cigarette use during the 8-week period via TLFB, as well as continued product use and quit attempts at the 12-week follow-up. We will explore differences in continued cigarette and e-cigarette use during the follow-up period and reported quit attempts between groups. We will explore differences in e-cigarette use, including flavors used, between groups as measures of acceptability of the product.

### Data collection, monitoring, and statistical plan

#### Data collection and storage

The proposed study will follow HIPAA guidelines for data collection, management, and monitoring to ensure the protection of participant information. Self-report data will be collected via Yale Qualtrics secure online software to ensure standardized assessments. Participant data will be coded by a unique subject identification number that does not contain any personal identifiers and data will be stored on a password-protected study computer. The key that links the participant to their unique subject identification and research records and any other electronic records (e.g., screening information and contact information) will be stored in a locked file on the encrypted study computer. De-identified data will be stored for analysis and will be available for research purposes to qualified individuals within the scientific community by request from the PI or through an NIH-funded repository.

#### Data safety monitoring plan

In addition to the oversight of the Yale Investigational Review Board (IRB), this project will use the existing Yale TCORS Data Safety Monitoring Board (DSMB) to provide the highest protection for study participants. The composition of the DSMB has been constructed following NIH guidelines and includes individuals with complementary expertise in tobacco use and statistics. The purview of the DSMB includes, but is not limited to, the following areas: assessments of data quality and timeliness, participant recruitment, retention, safety and efficacy data, and protocol compliance.

#### Data analysis plan

Data analyses will be conducted under the supervision of the study biostatistician at the end of the trial. No interim analyses are planned. Data on quantitative outcomes will be examined for conformity to the normal distribution, and if an outcome is not normally distributed, we have several options including applying transformations or utilizing alternative methods (e.g., generalized linear mixed models, nonparametric tests). For smoking outcomes that may be best modeled as count data, we can use generalized linear mixed models with Poisson or negative binomial distributions.

The primary analyses will be based on mixed models which use all available data on individuals, are flexible in accounting for the correlation structure among observations on the same individual and provide unbiased and efficient estimates when the data are missing at random. This approach, therefore, helps to avoid imputing missing data. The mixed model allows us to obtain unbiased and efficient estimates of change over time and between group differences. We will also perform sensitivity analyses based on pattern-mixture models to evaluate the robustness of our results to missing data assumptions.

#### Sample size and power calculations

Power was calculated using PASS software based on effect size estimates from pilot data where we detected an average change of 2.2 cigarettes, Cohen’s d = 0.68 [19]. To be sufficiently powered to detect a similar or smaller effect in the proposed study, we need *N* = 40 per group to achieve 80% power at two-sided alpha level of 0.05. Therefore, we will recruit 50 individuals per group for a total of 150 to allow up to 20% attrition and still achieve the specified power and required sample size of 40 per group. Due to our retention efforts, we expect our attrition rate may be even lower, as attrition in the initial study was less than the estimated 20% (i.e., attrition was 13%). For Aim 1, we will have sufficient power to detect a medium-to-large effect size (Cohen’s d = 0.64, Cohen’s f = 0.32). This corresponds to the ability to detect a difference of at least 2 cigarettes per day (SD = 3) or at least a 13% difference in percent days smoke-free (SD = 20%), consistent with effect size estimates from our work and other studies [19, 33]. For Aim 2, data from our initial work indicated differences across racial groups with a large effect size estimate (Cohen’s d = 1.21) [19]. With the proposed sample size, we will have sufficient power to detect a similar or smaller effect in the range of medium-to-large effect sizes (Cohen’s d = 0.64, Cohen’s f = 0.32) with 80% power at two-sided alpha level of 0.05 for the interactions of interest.

#### Analysis plan aim 1

**Examine the impact of prohibiting menthol flavor in cigarettes and e-cigarettes on smoking behavior**. We will use mixed model analyses to evaluate changes in smoking behavior from Phase I (usual menthol cigarettes) to Phase II (assigned regulatory scenarios). Outcomes were selected to inform the impact of these policies on public health including changes in cigarette use, addiction, and appeal/acceptability of the alternative product. The primary outcome will evaluate changes in the number of cigarettes smoked per day during the 8-week period and will examine differences by regulatory scenario. Regulatory scenario (i.e., no menthol restriction, menthol prohibited in cigarettes only, menthol prohibited in cigarettes and e-cigarettes) will be a between-subject factor and time (week 0, 2, 4, 6, 8) will be a within-subject factor. The best-fitting variance-covariance matrix will be selected based on Schwartz Bayesian Criterion (BIC). We hypothesize (1) greater reductions in cigarette use in the context of menthol flavor prohibited in cigarettes vs. no menthol cigarette restrictions and (2) the greatest reductions in cigarettes per day in the scenario where menthol is prohibited in cigarettes but where menthol e-cigarettes remain available to substitute for cigarettes. We will use focused contrasts to compare the average change in cigarette use between groups to evaluate the effect of prohibiting menthol flavor in cigarettes, e-cigarettes, or both products. We will also estimate the average change in each group by 95% confidence intervals. Secondary outcomes will compare percent days smoke-free between regulatory scenarios, as well as changes in nicotine dependence, motivation and confidence quitting smoking, and intentions to quit smoking between groups using the same approach. Exploratory outcomes will compare percent days of e-cigarette use during the 8-week period, rates of continued product use after products are no longer provided, and quit attempts during the follow-up period to evaluate differences by group. We will use 0.05 significance level for the primary analysis of each aim and Bonferroni-Holm correction for multiple secondary and exploratory analyses. Exploratory analyses of sex as a potential predictive and moderating factor will also be performed by including sex as an additional between-subject factor in the models. We do not have *a priori* hypotheses regarding sex effects in this study and will focus on providing sex-specific effect estimates to inform future studies.

#### Analysis plan aim 2

**Investigate whether outcomes differ by race to understand the impact of the menthol policies on Black individuals given high rates of menthol cigarette use in this population**. Consistent with our initial work, we aim to recruit equal numbers of participants who identify as Black vs. non-Black and will stratify randomization by race. This will allow us to evaluate the impact of the various regulatory scenarios separately by race and to understand the potential benefit specifically for Black individuals who smoke, who are disproportionately impacted by the harms of menthol cigarette use. We will examine whether changes in the outcomes differ by race by including race as an additional between-subject factor in the models above. We will test race*regulatory scenario*time interactions to evaluate moderator effects and race*time interactions to evaluate race as a predictor of outcome. Planned post-hoc analyses will evaluate pairwise differences in changes from Phase I to Phase II to compare the magnitude of the effect of the policy scenarios separately by race (e.g., the reduction in cigarette use for Black participants if menthol is prohibited in cigarettes only vs. both cigarettes and e-cigarettes vs. no policy change). If the findings suggest important differences in the magnitude of the effect between Black vs. non-Black individuals, this can also inform future research aimed at examining effects among specific subgroups by race/ethnicity.

### Dissemination plan

Study results will be disseminated through multiple outlets such as posting on Clinicaltrials.gov, presentations at scientific meetings, and publications in peer-reviewed journals. Each author will have participated sufficiently in the planning and contribution of the work, writing or revising of the draft, and final approval of the version to be submitted. As specified in the funding announcement, results will be made available to the FDA to inform the regulation of the manufacture, distribution, and marketing of tobacco products to protect public health. Additionally, study results will be disseminated into the community of study as well, such as through partnership with the Yale YCCI Cultural Ambassadors.

## Discussion

This study will be the first large-scale randomized clinical trial to evaluate the effect of potential policies prohibiting menthol flavor in cigarettes, e-cigarettes, or both products among adults who currently smoke menthol cigarettes. This study builds on earlier work estimating the potential effects of menthol flavor policies with simulation models by examining the effects of these policies on real-world smoking behavior among adults, including among Black adults who are impacted disproportionately by the health consequences of menthol cigarette use.

This research is being conducted in the context of a rapidly changing tobacco product landscape. Therefore, protocol changes may need to be made in response to changes in federal or state policies regarding menthol cigarettes or flavored e-cigarettes. For example, in the event that menthol is prohibited in cigarettes during the course of this project, we will still be able to provide novel data to inform the regulation of flavors in e-cigarettes which is an important area of study in tobacco regulatory science. In this case, we would plan to recruit individuals who had previously been smoking menthol cigarettes but switched to a different cigarette and will continue randomizing participants to the two regulatory scenarios that model either (1) prohibiting menthol in cigarettes only (i.e., non-menthol cigarettes with menthol flavored e-cigarettes available) or (2) prohibiting menthol in both cigarettes and e-cigarettes (i.e., non-menthol cigarettes with tobacco flavored e-cigarettes available). This alternative solution will still provide valuable information about the impact of the availability of menthol (vs. tobacco) flavored e-cigarettes on reductions in cigarette use. We will still be adequately powered to make comparisons between these scenarios, and by shifting to 2 scenarios while retaining our total target enrollment, we will have higher numbers of individuals in these groups than planned which could allow us to evaluate additional moderating factors by subgroups, such as sex or age. To identify potential moderating factors, we will compare randomized individuals before and after the enforcement of policies prohibiting menthol flavor in cigarettes to evaluate whether there is a shift in characteristics in our sample and will explore such factors as potential moderating effects for comparisons between the two regulatory scenarios. Furthermore, if there are changes in the available flavors in commercially available e-cigarettes used for this study, we will work with the FDA to identify appropriate products to address the study aims and apply for use of menthol and tobacco flavored e-cigarettes through an Investigational Tobacco Product application for research.

This project will provide critical information to understand whether continued availability of menthol (vs. tobacco) flavor in e-cigarettes will lead to greater reductions in cigarette use and complete switching to e-cigarettes following a policy prohibiting menthol cigarettes. Additionally, this study will investigate whether outcomes differ by race to understand the impact of the menthol policies on Black vs. non-Black individuals given high rates of menthol cigarette use among Black adults who smoke. Findings will provide timely and critical evidence regarding menthol flavor in cigarettes and e-cigarettes to inform regulatory actions to maximize reductions in the use of combustible cigarettes and reduce tobacco-related health disparities.

## Data Availability

No datasets were generated or analysed during the current study.

## References

[1] USDHHS. The health consequences of smoking—50 years of progress: a report of the Surgeon General. Atlanta, GA: US Department of Health and Human Services. Volume 17. Centers for Disease Control and Prevention, National Center for Chronic Disease Prevention and Health Promotion, Office on Smoking and Health; 2014.

[2] World Health Organization. WHO global report: Mortality attributable to tobacco. 2012.

[3] The NSDUH, Report. Recent Trends in Menthol Cigarette Use [press release]. November 18, 2011 2011.

[4] Villanti AC, Mowery PD, Delnevo CD, Niaura RS, Abrams DB, Giovino GA (2016). Changes in the prevalence and correlates of menthol cigarette use in the USA, 2004–2014. Tob Control.

[5] Smith SS, Fiore MC, Baker TB (2014). Smoking cessation in smokers who smoke menthol and non-menthol cigarettes. Addiction.

[6] Levy DT, Blackman K, Tauras J, Chaloupka FJ, Villanti AC, Niaura RS (2011). Quit attempts and quit rates among menthol and nonmenthol smokers in the United States. Am J Public Health.

[7] Ho JY, Elo IT (2013). The contribution of smoking to black-white differences in US mortality. Demography.

[8] World Health Organization Study Group on Tobacco Product Regulation (2016). Advisory note: banning Menthol in Tobacco products.

[9] Kyriakos CN, Driezen P, Fong G, Chung-Hall J, Hyland A, Geboers C et al. Impact of the European Union’s menthol cigarette ban on smoking cessation outcomes: longitudinal findings from the 2020–2021 ITC Netherlands surveys. Tob Control. 2022. 10.1136/tc-2022-057428 PMC11041602 36163172

[10] Chung-Hall J, Fong GT, Meng G, Cummings KM, Hyland A, O’Connor RJ (2022). Evaluating the impact of menthol cigarette bans on cessation and smoking behaviours in Canada: longitudinal findings from the Canadian arm of the 2016–2018 ITC Four Country smoking and vaping surveys. Tob Control.

[11] Tobacco. product standard for menthol in cigarettes, 87 FR 26454 (2022).

[12] Tobacco Products Scientific Advisory Committee (2011). Menthol cigarettes and public health: review of the scientific evidence and recommendations.

[13] FDA (2013). Preliminary scientific evaluation of the possible public health effects of menthol versus nonmenthol cigarettes.

[14] O’Connor RJ, Bansal-Travers M, Carter LP, Cummings KM (2012). What would menthol smokers do if menthol in cigarettes were banned? Behavioral intentions and simulated demand. Addiction.

[15] Chaiton M, Schwartz R, Cohen JE, Soule E, Eissenberg T (2018). Association of Ontario’s ban on menthol cigarettes with smoking behavior 1 month after implementation. JAMA Intern Med.

[16] Levy DT, Pearson JL, Villanti AC, Blackman K, Vallone DM, Niaura RS (2011). Modeling the future effects of a menthol ban on smoking prevalence and smoking-attributable deaths in the United States. Am J Public Health.

[17] Chaiton MO, Nicolau I, Schwartz R, Cohen JE, Soule E, Zhang B et al. Ban on menthol-flavoured tobacco products predicts cigarette cessation at 1 year: a population cohort study. Tob Control. 2019:tobaccocontrol–2018. 10.1136/tobaccocontrol-2018-054841 PMC6884656 31147474

[18] Cadham CJ, Sanchez-Romero LM, Fleischer NL, Mistry R, Hirschtick JL, Meza R (2020). The actual and anticipated effects of a menthol cigarette ban: a scoping review. BMC Public Health.

[19] Bold KW, Jatlow P, Fucito LM, Eid T, Krishnan-Sarin S, O’Malley S (2020). Evaluating the effect of switching to non-menthol cigarettes among current menthol smokers: an empirical study of a potential ban of characterising menthol flavour in cigarettes. Tob Control.

[20] Tobacco product standard for characterizing flavors in cigars, 87 FR 26396. (2022).

[21] FDA. Deeming Tobacco Products To Be Subject to the Federal Food (2016). Drug, and Cosmetic Act, as amended by the Family Smoking Prevention and Tobacco Control Act; restrictions on the sale and distribution of Tobacco products and Required Warning Statements for Tobacco Products. Final rule. Fed Regist.

[22] FDA finalizes enforcement policy on unauthorized flavored cartridge-based. e-cigarettes that appeal to children, including fruit and mint [press release]. 2020.

[23] Administration FaD. Enforcement priorities for Electronic Nicotine Delivery Systems (ENDS) and Other Deemed Products on the Market Without Premarket Authorization (Revised). 2020. Report No.: FDA-2019-D-0661.

[24] Hung F, Wallach JD, O’Malley SS, Bold KW (2021). Characteristics of registered clinical trials evaluating the role of e-Cigarettes in Cessation or reduction of cigarette smoking. JAMA Psych.

[25] Cornelius ME, Loretan CG, Jamal A, Lynn BCD, Mayer M, Alcantara IC (2023). Tobacco product use among adults–United States, 2021. Morb Mortal Wkly Rep.

[26] Gades MS, Alcheva A, Riegelman AL, Hatsukami DK. The role of nicotine and flavor in the abuse potential and appeal of electronic cigarettes for adult current and former cigarette and electronic cigarette users: a systematic review. Nicotine Tob Res. 2022:1–12. 10.1093/ntr/ntac073 PMC9356694 35305014

[27] Hajek P, Pittaccio K, Pesola F, Myers Smith K, Phillips-Waller A, Przulj D (2020). Nicotine delivery and users’ reactions to Juul compared with cigarettes and other e‐cigarette products. Addiction.

[28] Goniewicz ML, Gawron M, Smith DM, Peng M, Jacob P, Benowitz NL (2017). Exposure to nicotine and selected toxicants in cigarette smokers who switched to electronic cigarettes: a longitudinal within-subjects observational study. Nicotine Tob Res.

[29] Lindson N, Butler AR, McRobbie H, Bullen C, Hajek P, Begh R et al. Electronic cigarettes for smoking cessation. Cochrane Database Syst Rev. 2024(1). 10.1002/14651858.CD010216.pub4 PMC8094228 33052602

[30] Bold KW, Buta E, Simon P, Gueorguieva R, Jackson A, Suttiratana SC et al. Examining the potential role of e-cigarettes to reduce health disparities associated with menthol cigarette use: characterizing e-cigarette use, flavors, and reasons for use among US adults smoking menthol cigarettes. Drug Alcohol Depend. 2022:109475. 10.1016/j.drugalcdep.2022.109475 PMC9248755 35594642

[31] Hooper MW, Smiley SL (2018). Comparison of e-cigarette use among menthol and non-menthol smokers: findings from a community based sample. Ethn Dis.

[32] Kotlyar M, Shanley R, Dufresne SR, Corcoran GA, Hatsukami DK (2022). Effect on Tobacco Use and subjective measures of including E-cigarettes in a simulated Ban of Menthol in Combustible cigarettes. Nicotine Tob Res.

[33] Hatsukami DK, Meier E, Lindgren BR, Anderson A, Reisinger SA, Norton KJ (2020). A randomized clinical trial examining the effects of instructions for electronic cigarette use on smoking-related behaviors and biomarkers of exposure. Nicotine Tob Res.

[34] Donny EC, Denlinger RL, Tidey JW, Koopmeiners JS, Benowitz NL, Vandrey RG (2015). Randomized trial of reduced-nicotine standards for cigarettes. N Engl J Med.

[35] Hatsukami DK, Kotlyar M, Hertsgaard LA, Zhang Y, Carmella SG, Jensen JA (2010). Reduced nicotine content cigarettes: effects on toxicant exposure, dependence and cessation. Addiction.

[36] Mercincavage M, Souprountchouk V, Tang KZ, Dumont RL, Wileyto EP, Carmella SG et al. A randomized controlled trial of progressively reduced nicotine content cigarettes on smoking behaviors, biomarkers of exposure, and subjective ratings. Cancer Epidemiol Biomarkers Prev. 2016:cebp. 1088.2015. 10.1158/1055-9965.EPI-15-1088 PMC4930705 27197288

[37] Huang J, Duan Z, Kwok J, Binns S, Vera LE, Kim Y (2019). Vaping versus JUULing: how the extraordinary growth and marketing of JUUL transformed the US retail e-cigarette market. Tob Control.

[38] Conway J. Vaping market share in the United States in 2020, by brand. 2020.

[39] Nardone N, Helen GS, Addo N, Meighan S, Benowitz NL (2019). JUUL electronic cigarettes: Nicotine exposure and the user experience. Drug Alcohol Depend.

[40] Brown J, DeAtley T, Welding K, Schwartz R, Chaiton M, Lawrence Kittner D (2017). Tobacco industry response to menthol cigarette bans in Alberta and Nova Scotia, Canada. Tob Control.

[41] Schwartz R, Chaiton M, Borland T, Diemert L (2018). Tobacco industry tactics in preparing for menthol ban. Tob Control.

[42] Brown RA, Burgess ES, Sales SD, Whiteley JA, Evans DM, Miller IW (1998). Reliability and validity of a smoking timeline follow-back interview. Psychol Addict Behav.

[43] Edelen MO, Huang W, Stucky BD (2016). Additional validity evidence for the PROMIS smoking assessment toolkit. Addict Behav.

[44] Edelen MO, Tucker JS, Shadel WG, Stucky BD, Cai L (2012). Toward a more systematic assessment of smoking: development of a smoking module for PROMIS®. Addict Behav.

[45] Morean M, Krishnan-Sarin S, Sussman S, Foulds J, Fishbein H, Grana R (2019). Psychometric evaluation of the E-cigarette dependence scale. Nicotine Tob Res.

[46] Etter JF, Sutton S (2002). Assessing ‘stage of change’in current and former smokers. Addiction.

[47] Spitzer RL, Kroenke K, Williams JB, Löwe B (2006). A brief measure for assessing generalized anxiety disorder: the GAD-7. Arch Intern Med.

[48] Kroenke K, Spitzer RL (2002). The PHQ-9: a new depression diagnostic and severity measure. Psychiatric Annals.

[49] Stevanovic D (2011). Quality of life enjoyment and satisfaction questionnaire–short form for quality of life assessments in clinical practice: a psychometric study. J Psychiatr Ment Health Nurs.

[50] Williams MT, Printz D, DeLapp RC (2018). Assessing racial trauma with the trauma symptoms of discrimination scale. Psychol Violence.

[51] Phinney JS (1992). The multigroup ethnic identity measure: a new scale for use with diverse groups. J Adolesc Res.

[52] Brown SD, Unger Hu KA, Mevi AA, Hedderson MM, Shan J, Quesenberry CP (2014). The Multigroup ethnic identity Measure—Revised: measurement invariance across racial and ethnic groups. J Couns Psychol.

[53] Shadel WG, Edelen MO, Tucker JS, Stucky BD, Hansen M, Cai L (2014). Development of the PrOMis® nicotine dependence item banks. Nicotine Tob Res.

[54] Piper ME, Piasecki TM, Federman EB, Bolt DM, Smith SS, Fiore MC (2004). A multiple motives approach to tobacco dependence: the Wisconsin Inventory of Smoking Dependence motives (WISDM-68). J Consult Clin Psychol.

[55] Smith SS, Piper ME, Bolt DM, Fiore MC, Wetter DW, Cinciripini PM (2010). Development of the brief Wisconsin inventory of smoking dependence motives. Nicotine Tob Res.

[56] Heatherton TF, Kozlowski LT, Frecker RC, Fagerstrom K (1991). The Fagerström test for nicotine dependence: a revision of the Fagerstrom Tolerance Questionnaire. Br J Addict.

[57] Cappelleri JC, Bushmakin AG, Baker CL, Merikle E, Olufade AO, Gilbert DG (2007). Confirmatory factor analyses and reliability of the modified cigarette evaluation questionnaire. Addict Behav.

[58] Morean ME, Bold KW, The Modified E-cigarette Evaluation questionnaire: psychometric evaluation of an adapted version of the modified cigarette evaluation questionnaire for Use with adults who Use Electronic Nicotine Delivery systems. Nicotine Tob Res. 2022; 24(9), 1396–1404. 10.1093/ntr/ntac062 PMC9356680 35271732

[59] West R, Hajek P (2004). Evaluation of the mood and physical symptoms scale (MPSS) to assess cigarette withdrawal. Psychopharmacology.

[60] Toll BA, O’Malley SS, McKee SA, Salovey P, Krishnan-Sarin S (2007). Confirmatory factor analysis of the Minnesota nicotine withdrawal scale. Psychol Addict Behav.

